# Incidence of remission and relapse of proteinuria, end-stage kidney disease, mortality, and major outcomes in primary nephrotic syndrome: the Japan Nephrotic Syndrome Cohort Study (JNSCS)

**DOI:** 10.1007/s10157-020-01864-1

**Published:** 2020-03-07

**Authors:** Ryohei Yamamoto, Enyu Imai, Shoichi Maruyama, Hitoshi Yokoyama, Hitoshi Sugiyama, Kosaku Nitta, Tatsuo Tsukamoto, Shunya Uchida, Asami Takeda, Toshinobu Sato, Takashi Wada, Hiroki Hayashi, Yasuhiro Akai, Megumu Fukunaga, Kazuhiko Tsuruya, Kosuke Masutani, Tsuneo Konta, Tatsuya Shoji, Takeyuki Hiramatsu, Shunsuke Goto, Hirofumi Tamai, Saori Nishio, Arimasa Shirasaki, Kojiro Nagai, Kunihiro Yamagata, Hajime Hasegawa, Hideo Yasuda, Shizunori Ichida, Tomohiko Naruse, Tomoya Nishino, Hiroshi Sobajima, Satoshi Tanaka, Toshiyuki Akahori, Takafumi Ito, Yoshio Terada, Ritsuko Katafuchi, Shouichi Fujimoto, Hirokazu Okada, Eiji Ishimura, Junichiro J. Kazama, Keiju Hiromura, Tetsushi Mimura, Satoshi Suzuki, Yosuke Saka, Tadashi Sofue, Yusuke Suzuki, Yugo Shibagaki, Kiyoki Kitagawa, Kunio Morozumi, Yoshiro Fujita, Makoto Mizutani, Takashi Shigematsu, Naoki Kashihara, Hiroshi Sato, Seiichi Matsuo, Ichiei Narita, Yoshitaka Isaka

**Affiliations:** 1 Health and Counseling Center, Osaka University, 1-17 Machikaneyama-cho, Toyonaka, Osaka 560-0043 Japan; 2 Department of Nephrology, Osaka University Graduate School of Medicine, 2-2-D11 Yamadaoka, Suita, Osaka 565-0871 Japan; 3 Nakayamadera Imai Clinic, 2-8-18 Nakayamadera, Takarazuka, Hyogo 665-0861 Japan; 4 Department of Nephrology, Nagoya University Graduate School of Medicine, 65 Tsurumai-cho, Showa-ku, Nagoya, Aichi 466-8550 Japan; 5 Department of Nephrology, Kanazawa Medical Univeristy School of Medicine, 1-1 Daigaku, Uchinada, Kahoku, Ishikawa 920-0293 Japan; 6 Department of Nephrology, Rheumatology, Endocrinology and Metabolism, Okayama University Graduate School of Medicine, Dentistry and Pharmaceutical Sciences, 2-5-1 Shikatacho, Kita-ku, Okayama, Okayama 700-8558 Japan; 7 Department of Nephrology, Tokyo Women’s Medical University, 8-1 Kawada-cho, Shinjuku-ku, Tokyo, 162-8666 Japan; 8 Department of Nephrology and Dialysis, Kitano Hospital, Tazuke Kofukai Medical Research Institute, 2-4-20 Ogimachi, Kita-ku, Osaka, Osaka 530-8480 Japan; 9 Department of Internal Medicine, Teikyo University School of Medicine, 2-11-1 Kaga, Itabashi-ku, Tokyo, 173-8606 Japan; 10 Kidney Disease Center, Japanese Red Cross Nagoya Daini Hospital, 2-9 Myokencho, Showa-ku, Nagoya, Aichi 466-8650 Japan; 11 Department of Nephrology, JCHO Sendai Hospital, 3-16-1 Tsutsumi-machi, Aoba-ku, Sendai, Miyagi 981-8501 Japan; 12 Department of Nephrology and Laboratory Medicine, Kanazawa University, 13-1 Takara-machi, Kanazawa, Ishikawa 920-8641 Japan; 13 Department of Nephrology, Fujita Health University School of Medicine, 1-98 Dengakugakubo, Kutsukakecho, Toyoake, Aichi 470-1192 Japan; 14 First Department of Internal Medicine, Nara Medical University, 840 Shijocho, Kashihara, Nara 634-8522 Japan; 15 Division of Nephrology, Department of Internal Medicine, Toyonaka Municipal Hospital, 4-14-1 Shibaharacho, Toyonaka, Osaka 560-8565 Japan; 16 Department of Integrated Therapy for Chronic Kidney Disease, Graduate School of Medical Sciences, Kyushu University, 3-1-1 Maidashi, Higashi-ku, Fukuoka, Fukuoka 812-8582 Japan; 17 Division of Nephrology and Rheumatology, Department of Internal Medicine, Faculty of Medicine, Fukuoka University, 7-45-1 Nanakuma, Jonan-ku, Fukuoka, Fukuoka 814-0180 Japan; 18 Department of Cardiology, Pulmonology, and Nephrology, Yamagata University School of Medicine, 2-2 Iida-Nishi, Yamagata-shi, Yamagata, Yamagata 990-9585 Japan; 19 Department of Kidney Disease and Hypertension, Osaka General Medical Center, 3-1-56 Bandaihigashi, Sumiyoshi-ku, Osaka, Osaka 558-8558 Japan; 20 Department of Nephrology, Konan Kosei Hospital, 137 Omatsubara, Takayacho, Konan, Aichi 483-8704 Japan; 21 Division of Nephrology and Kidney Center, Kobe University Graduate School of Medicine, 7-5-1 Kusunokicho, Cuho-ku, Kobe, Hyogo 650-0017 Japan; 22 Department of Nephrology, Anjo Kosei Hospital, 28 Higashihirokute, Anjocho, Anjo, Aichi 446-8602 Japan; 23 Division of Rheumatology, Endocrinology and Nephrology, Hokkaido University Graduate School of Medicine, Kita 15, Nishi 7, Kita-ku, Sapporo, Hokkaido 060-8638 Japan; 24 Department of Nephrology, Ichinomiya Municipal Hospital, 2-2-22 Bunkyo, Ichinomiya, Aichi 491-8558 Japan; 25 Department of Nephrology, Institute of Biomedical Sciences, Tokushima University Graduate School, 3-18-15, Kuramoto-cho, Tokushima, 770-8503 Japan; 26 Department of Nephrology, Faculty of Medicine, University of Tsukuba, 1-1-1 Tennodai, Tsukuba, Ibaraki 305-8575 Japan; 27 Department of Nephrology and Hypertension, Saitama Medical Center, Saitama Medical University, 1981 Kamoda, Kawagoe, Saitama 350-850 Japan; 28 Internal Medicine 1, Hamamatsu University School of Medicine, 1-20-1 Handayama, Higashi-ku, Hamamatsu, Shizuoka 431-3192 Japan; 29 Department of Nephrology, Japanese Red Cross Nagoya Daiichi Hospital, 3-35 Michishitacho, Nakamura-ku, Nagoya, Aichi 453-8511 Japan; 30 Department of Nephrology, Kasugai Municipal Hospital, 1-1-1 Takakicho, Kasugai, Aichi 486-8510 Japan; 31 Department of Nephrology, Nagasaki University Hospital, 1-7-1 Sakamoto, Nagasaki, Nagasaki 852-8501 Japan; 32 Department of Diabetology and Nephrology, Ogaki Municipal Hospital, 4-86 Minaminokawacho, Ogaki, Gifu 503-8502 Japan; 33 Department of Nephrology, Shizuoka General Hospital, 4-27-1 Kitaando, Aoi-ku, Shizuoka, Shizuoak 420-8527 Japan; 34 Department of Nephrology, Chutoen General Medical Center, 1-1 Shobugaike, Kakegawa, Shizuoka 436-8555 Japan; 35 Division of Nephrology, Shimane University Hospital, 89-1 Enyacho, Izumo, Shimane 693-8501 Japan; 36 Department of Endocrinology, Metabolism and Nephrology, Kochi Medical School, Kochi University, Okocho Kohasu, Nankoku, Kochi 783-8505 Japan; 37 Kideny Unit, National Hospital Organization Fukuokahigashi Medical Center, 1-1-1 Chidori, Koga, Fukuoka 811-3195 Japan; 38 Department of Hemovascular Medicine and Artificial Organs, Faculty of Medicine, University of Miyazaki, 5200 Kihara, Kiyotakecho, Miyazaki, Miyazaki 889-1692 Japan; 39 Department of Nephrology, Saitama Medical University, 38 Morohongo, Moroyama, Iruma, Saitama 350-0495 Japan; 40 Department of Nephrology, Osaka City University Graduate School of Medicine, 1-4-3 Asahimachi, Abeno-ku, Osaka, 545-8585 Japan; 41 Department of Nephrology and Hypertension, Fukushima Medical University School of Medicine, 1 Hikariga-oka, Fukushima-City, Fukushima 960-1295 Japan; 42 Department of Nephrology and Rheumatology, Gunma University Graduate School of Medicine, 3-39-22 Showa-matchi, Maebashi, Gunma 371-8511 Japan; 43 Department of Nephrology, Gifu Prefectural Tajimi Hospital, 5-161 Maebatacho, Tajimi, Gifu 507-8522 Japan; 44 Department of Nephrology, Kainan Hospital, 396 Minamihonden, Maegasucho, Yatomi, Aichi 498-8502 Japan; 45 Department of Nephrology, Yokkaichi Municipal Hospital, 2-2-37 Shibata, Yokkaichi, Mie 510-8567 Japan; 46 Department of Cardiorenal and Cerebrovascular Medicine, Kagawa University, 1750-1 Ikenobe, Miki-cho, Kita-gun, Kagawa 761-0793 Japan; 47 Division of Nephrology, Department of Internal Medicine, Juntendo University Faculty of Medicine, 2-1-1 Hongo, Bunkyo-ku, Tokyo, 113-8421 Japan; 48 Division of Nephrology and Hypertension, Department of Internal Medicine, St. Marianna University School of Medicine, 2-16-1 Sugao, Miyamae-ku, Kawasaki, Kanagawa 216-000 Japan; 49 Division of Internal Medicine, National Hospital Organization Kanazawa Medical Center, 1-1 Shimoishibikimachi, Kanazawa, Ishikawa 920-8650 Japan; 50 Department of Nephrology, Masuko Memorial Hospital, 35-28 Takegashicho, Nakamura-ku, Nagoya, Aichi 453-0016 Japan; 51 Department of Nephrology, Chubu Rosai Hospital, 1-10-6 Komei, Minato-ku, Nagoya, Aichi 455-8530 Japan; 52 Department of Nephrology, Handa City Hospital, 2-29 Toyocho, Handa, Aichi 475-8599 Japan; 53 Department of Nephrology, Wakayama Medical University, 811-1 Kimiidera, Wakayama-City, Wakayama 641-8509 Japan; 54 Department of Nephrology and Hypertension, Kawasaki Medical School, 577 Matsushima, Kurashiki, Osakayama 701-0192 Japan; 55 Department of Nephrology, Endocrinology, and Vascular Medicine, Tohoku Univeristy Gradaute School of Medicine, 1-1 Seiryo-cho, Aoba-ku, Sendai, Miyagi 980-8574 Japan; 56 Division of Clinical Nephrology and Rheumatology, Kidney Research Center, Niigata University Graduate School of Medical and Dental Sciences, 757 Ichibancho, Asahimachi-dori, Chuo Ward, Niigata, Niigata 951-8510 Japan

**Keywords:** Primary nephrotic syndrome, Cohort study, Mortality, End-stage kidney disease, Diabetes, Infection

## Abstract

**Background:**

Despite recent advances in immunosuppressive therapy for patients with primary nephrotic syndrome, its effectiveness and safety have not been fully studied in recent nationwide real-world clinical data in Japan.

**Methods:**

A 5-year cohort study, the Japan Nephrotic Syndrome Cohort Study, enrolled 374 patients with primary nephrotic syndrome in 55 hospitals in Japan, including 155, 148, 38, and 33 patients with minimal change disease (MCD), membranous nephropathy (MN), focal segmental glomerulosclerosis (FSGS), and other glomerulonephritides, respectively. The incidence rates of remission and relapse of proteinuria, 50% and 100% increases in serum creatinine, end-stage kidney disease (ESKD), all-cause mortality, and other major adverse outcomes were compared among glomerulonephritides using the Log-rank test. Incidence of hospitalization for infection, the most common cause of mortality, was compared using a multivariable-adjusted Cox proportional hazard model.

**Results:**

Immunosuppressive therapy was administered in 339 (90.6%) patients. The cumulative probabilities of complete remission within 3 years of the baseline visit was ≥ 0.75 in patients with MCD, MN, and FSGS (0.95, 0.77, and 0.79, respectively). Diabetes was the most common adverse events associated with immunosuppressive therapy (incidence rate, 71.0 per 1000 person-years). All-cause mortality (15.6 per 1000 person-years), mainly infection-related mortality (47.8%), was more common than ESKD (8.9 per 1000 person-years), especially in patients with MCD and MN. MCD was significantly associated with hospitalization for infection than MN.

**Conclusions:**

Patients with MCD and MN had a higher mortality, especially infection-related mortality, than ESKD. Nephrologists should pay more attention to infections in patients with primary nephrotic syndrome.

**Electronic supplementary material:**

The online version of this article (10.1007/s10157-020-01864-1) contains supplementary material, which is available to authorized users.

## Introduction

Nephrotic syndrome is characterized by massive proteinuria, edema, and hypoalbuminuria [1]. Epidemiological studies have shown that patients with nephrotic syndrome are vulnerable to a wide variety of adverse events: end-stage kidney disease (ESKD) [2–4], thromboembolism [5], infection [6], malignancy [7], cardiovascular disease (CVD) [8], and all-cause mortality [9]. Primary nephrotic syndrome is the major cause of nephrotic syndrome diagnosed using kidney biopsy, including mainly minimal change disease (MCD), membranous nephropathy (MN), and focal segmental glomerulosclerosis (FSGS) [10]. A systematic review reported that incidences rates of MCD, MN, and FSGS were 0.2–0.8, 0.3–1.4, and 0.2–1.1 per 100,000 person-years, respectively [11].

Immunosuppressive therapy is the main treatment modality for patients with primary nephrotic syndrome as suggested by the clinical guidelines of primary nephrotic syndromes [12, 13]. Systematic reviews of randomized controlled trials on immunosuppressive therapy in patients with MN, the most extensively studied glomerulonephritis in primary nephrotic syndrome, clarified that some immunosuppressive drugs reduced all-cause mortality and risk of ESKD, although the number of trials with a high-quality design was relatively small and most trials did not have adequate follow-up and enough power to assess the prespecified definite outcomes [14, 15]. These systematic reviews also suggested that the drugs were associated with substantial toxicity leading to withdrawals or hospitalization. Their results potentially underestimated the toxicity of immunosuppressive therapy in the real world because patients with a higher risk of toxicity, such as elderly patients, are often excluded in randomized trials [16]. To establish the treatment strategy that has a high effectiveness and low risk of adverse effects, an observational study using real-world data, including patients with a high risk of toxicity from therapeutic interventions, is essential.

The aim of the present cohort study, the Japan Nephrotic Syndrome Cohort Study (JNSCS) [17], was to clarify the incidence of major clinical outcomes in 374 patients with primary nephrotic syndrome during the 5-year follow-up period. The outcomes of interest were remission and relapse of proteinuria, deterioration in kidney function (50% and 100% increases in serum creatinine level and ESKD), CVD, all-cause mortality, and other adverse events associated with immunosuppressive therapy, including infection, diabetes, arteriovenous thrombosis, aseptic osteonecrosis, and peptic ulcers. The results of the present study provide pivotal information to determine the clinical goals of the treatments for primary nephrotic syndrome.

## Materials and methods

### Participants

**Fig. 1:**
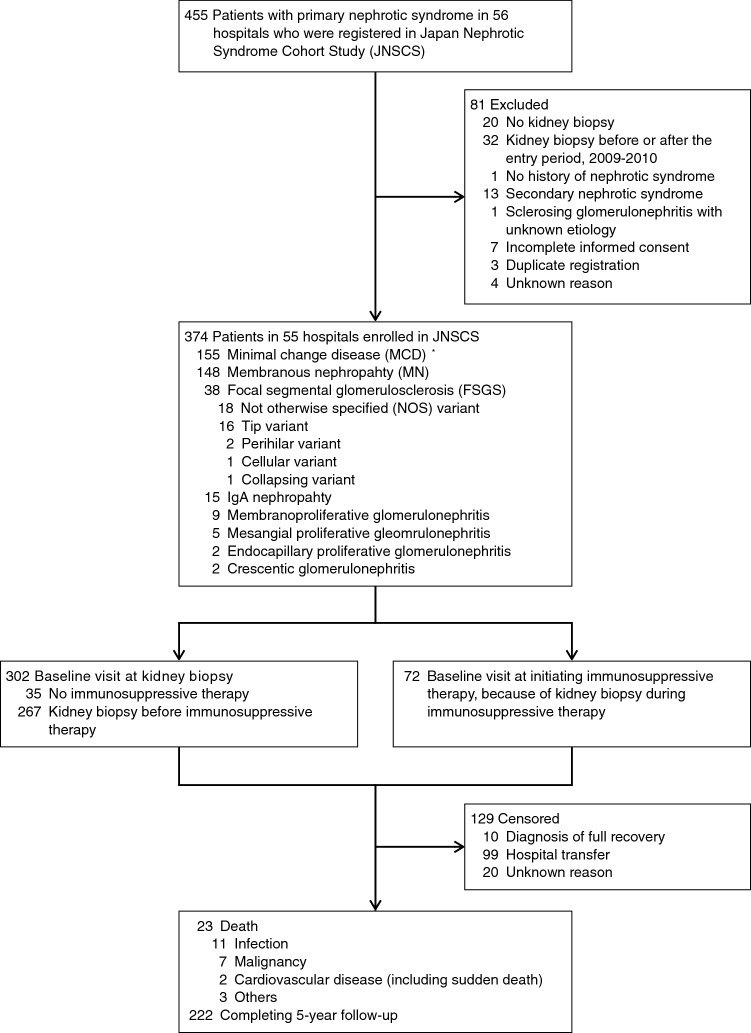
Flow diagram of patients in the Japan Nephrotic Syndrome Cohort Study (JNSCS). *Including two patients who were diagnosed with MCD at the first kidney biopsy but re-diagnosed with FSGS (NOS variant) at the second biopsy 33 and 1344 days after the first biopsy

The JNSCS is a 5-year multicenter cohort study of primary nephrotic syndrome to clarify the incidence rates of major clinical outcomes and assess the effectiveness of immunosuppressive therapy in Japan. Details of the study design was described elsewhere [17]. Briefly, 455 nephrotic patients were registered in the JNSCS, who were diagnosed with primary nephrotic syndrome using kidney biopsy during the entry period between January 2009 and December 2010 in 56 hospitals (Fig. 1). The diagnosis of primary nephrotic syndrome was based on the clinical and histopathological characteristics [18]. Nephrotic patients with minor glomerular abnormalities by light microscopy was diagnosed as MCD. The diagnosis of MN was made by the detection of granular deposits of mainly IgG along the glomerular capillary walls by immunofluorescence microscopy with or without thickening of the glomerular capillary wall by light microscopy. FSGS included five variants: collapsing, tip, cellular, perihilar, and not-otherwise specified (NOS) variants [19]. After excluding 81 patients with no kidney biopsy (*N* = 20), kidney biopsy before or after the entry period (*N* = 32), no history of nephrotic syndrome (*N* = 1), diagnosis of secondary nephrotic syndrome (*N* = 13), sclerosing glomerulonephritis with unknown etiology (*N* = 1), incomplete informed consent (*N* = 7), duplicate registrations (*N* = 3) and unknown reason (*N* = 4), 374 patients with primary nephrotic syndrome in 55 hospitals were finally enrolled in JNSCS, including those with MCD (*N* = 155), MN (*N* = 148), FSGS (*N* = 38), IgA nephropathy (*N* = 15), membranoproliferative glomerulonephritis (*N* = 9), mesangial proliferative glomerulonephritis (*N* = 5), endocapillary proliferative glomerulonephritis (*N* = 2), and crescentic glomerulonephritis (*N* = 2). Because of the small number of patients with glomerulonephritides except those with MCD, MN, and FSGS, the patients were classified into four groups of glomerulonephritides: MCD, MN, FSGS, and other glomerulonephritides.

The study protocol of JNSCS was approved by the ethics committee of Osaka University Hospital (approval number 17035-4) and the institutional review board of each participating hospital. All procedures performed in the present study were in accordance with the World Medical Association Declaration of Helsinki.

### Measurements

The clinical characteristics at the kidney biopsy and, if immunosuppressive therapy was administered, those at initiating immunosuppressive therapy were collected in JNSCS, including age, sex, body mass index, systolic and diastolic blood pressure, 24-h urinary protein, urinary protein-to-creatinine ratio, serum concentration of creatinine, albumin, and total cholesterol, hemoglobin A1c, and use of renin-angiotensin system (RAS) blockers, statins, and antidiabetic drugs. To calculate the estimated glomerular filtration rate (eGFR) in adult patients aged 18 years or older, the Japanese equation was used: eGFR = 194 × age (year)^−0.287^ × serum creatinine (mg/dL)^−1.094^ × 0.739 (if female) [20]. As a measure of the baseline urinary protein, 24-h urinary protein was preferred. Urinary protein-to-creatinine ratio was alternatively used only in patients with missing values of the baseline 24-h urinary protein. In the present study, the baseline visit was set at the kidney biopsy or the first date of immunosuppressive therapy, whichever came first.

The outcome measures of interest in the present study consisted of the time to remission and relapse of proteinuria; 50% and 100% irreversible increases in serum creatinine level; ESKD requiring kidney replacement therapy; use of antidiabetic drugs; hospitalization for infection, CVD, and arteriovenous thrombosis; diagnosis of malignancy, aseptic osteonecrosis, and peptic ulcer; and all-cause mortality. Remission of proteinuria was categorized into complete remission, incomplete remission type 1, and incomplete remission type 2; complete remission was defined as 24-h urinary protein of < 0.3 g/day or urinary protein-to-creatinine ratio of < 0.3 g/gCr; incomplete remission type 1 was defined as 24-h urinary protein of < 1.0 g/day or urinary protein-to-creatinine ratio of < 1.0 g/gCr; incomplete remission type 2 was defined as 24-h urinary protein of < 3.5 g/day or urinary protein-to-creatinine ratio of < 3.5 g/gCr [2, 13]. Relapse of proteinuria was defined as 24-h urinary protein of ≥ 1.0 g/day, urinary protein-to-creatinine ratio of ≥ 1.0 g/gCr, and/or 2 + or more of positive dipstick tests for urinary protein continued two times or more in patients with complete remission [13]. CVD included heart disease, stroke, peripheral arterial disease, and sudden death. In patients followed up for more than 5 years, the end of the follow-up was set at 5 years after the baseline visit of each patient. Patients who died were regarded as censored, except in the analyses of all-cause mortality (Supplementary Fig. 1A and B).

### Statistical analyses

Baseline clinical characteristics among the four groups of glomerulonephritides were compared using the chi-square test, Fisher's exact test, ANOVA, or Kruskal–Wallis test, as appropriate.

To compare the incidence rates of each outcome among the four groups of glomerulonephritides, their cumulative probabilities were estimated using the Kaplan–Meier method and compared using the Log-rank test. The cumulative probabilities of complete remission, incomplete remission type 1, and incomplete remission type 2 were calculated in 292, 367, and 370 patients with baseline urinary protein of ≥ 0.3, ≥ 1.0, and ≥ 3.5 g/day (or g/gCr), respectively (Supplementary Fig. 1A). To calculate those of relapse of proteinuria after complete remission, 290 patients who achieved complete remission and were followed up thereafter were included. After excluding 16 patients with the baseline use of diabetic drugs, 358 patients with no baseline use of antidiabetic drugs were included for calculation of the cumulative probability of use of diabetic drugs (Supplementary Fig. 1B). The incidence rate of each outcome was calculated based on the Poisson distribution and expressed as the number of events per 1000 person-years.

Because infection was the leading cause of mortality, the incidence of hospitalization for infection was compared among the four groups of glomerulonephritides using unadjusted and multivariable-adjusted Cox proportional hazards models. The proportional hazards assumption for covariates was checked using Schoenfeld residuals. Because the proportional hazards assumption of sex was violated, all multivariable-adjusted Cox proportional hazards models were stratified according to sex to control its potential confounding effect. Multivariable-adjusted model 1 included age as covariates. Models 2 and 3 included serum creatinine and urinary protein as covariates in an additive manner.

Normally distributed continuous variables are expressed as mean ± standard deviation, and non-normally distributed continuous variables as median (interquartile range). Categorical variables are expressed as numbers (proportions). *P* < 0.05 was considered statistically significant. All statistical analyses were performed using R version 3.6.0 (The R Foundation for Statistical Computing, https://www.r-project.org/).

## Results

The clinical characteristics of 155 (41.4%), 148 (39.6%), 38 (10.2%), and 33 (8.8%) patients with MCD, MN, FSGS, and others, respectively, are listed in Table 1. The baseline visit was set at the beginning of immunosuppressive therapy in 45 (29.0%), 14 (9.5%), 9 (23.7%), and 4 (12.1%) patients with MCD, MN, FSGS, and others, respectively, because they underwent kidney biopsy after initiating immunosuppressive therapy, whereas it was set at the date of kidney biopsy in the remaining patients. At their baseline visits, significant differences among the four groups were observed in terms of the age, body mass index, systolic and diastolic blood pressure, urinary protein, serum creatinine, eGFR, serum albumin, serum total cholesterol, and use of RAS blockers, statin, and antidiabetic drugs (*P* < 0.05). Patients with MCD were likely to have received immunosuppressive therapy before kidney biopsy and have a younger age, higher levels of body mass index, eGFR, and serum total cholesterol, lower levels of blood pressure, serum creatinine and serum albumin, and lower proportion of use of RAS blockers, compared with that in patients with MN, FSGS, and others.

**Table 1:** Clinical characteristics of 374 patients with primary nephrotic syndrome

	MCD	MN	FSGS	Others
*N*	155	148	38	33
Baseline visit, *N* (%)^*a^				
Kidney biopsy	110 (71.0)	134 (90.5)	29 (76.3)	29 (87.9)
Immunosuppressive therapy	45 (29.0)	14 (9.5)	9 (23.7)	4 (12.1)
Clinical characteristics at baseline visit				
Age (year)^*^	41 (26, 61)	66 (59, 74)	62 (29, 73)	58 (46, 71)
< 18 years, *N* (%)	16 (10.3)	1 (0.7)	0 (0.0)	1 (3.0)
Male, *N *(%)	90 (58.1)	83 (56.1)	25 (65.8)	19 (57.6)
Body mass index (kg/m^2^)^*b^	24.1 ± 4.3	23.8 ± 3.5	23.5 ± 3.8	23.1 ± 3.5
Systolic blood pressure (mmHg)^*b^	121 ± 17	131 ± 20	135 ± 18	136 ± 14
Diastolic blood pressure (mmHg)^*b^	73 ± 12	77 ± 13	79 ± 13	77 ± 11
Urinary protein (g/day) (or g/gCr)^*bc^	6.8 (4.8, 10.4)	4.4 (2.9, 6.3)	7.5 (4.5, 10.7)	5.1 (3.4, 6.9)
≥ 3.5 g/day (or g/gCr), *N* (%)	133 (86.4)	101 (68.7)	35 (92.1)	24 (72.7)
1.0–3.4	19 (12.3)	43 (29.3)	3 (7.9)	9 (27.3)
0.3–0.9	0 (0.0)	3 (2.0)	0 (0.0)	0 (0.0)
< 0.3	2 (1.3)	0 (0.0)	0 (0.0)	0 (0.0)
Serum creatinine (mg/dL)^*^	0.87 (0.70, 12.0)	0.87 (0.87, 1.56)	1.11 (0.87, 1.56)	1.04 (0.82, 1.50)
eGFR, mL/min/1.73 m^2*b^	68 ± 27	61 ± 21	52 ± 22	52 ± 25
≥ 90 mL/min/1.73 m^2^, *N* (%)	23 (16.5)	12 (8.2)	1 (2.6)	3 (9.4)
60–89	61 (43.9)	69 (46.9)	13 (34.2)	9 (28.1)
45–59	28 (20.1)	38 (25.9)	11 (28.9)	7 (21.9)
30–44	16 (11.5)	15 (10.2)	4 (10.5)	5 (15.6)
15–29	7 (5.0)	10 (6.8)	7 (18.4)	7 (21.9)
< 15	4 (2.9)	3 (2.0)	2 (5.3)	1 (3.1)
Serum albumin (g/dL)^*^	1.7 ± 0.6	2.2 ± 0.6	1.9 ± 0.7	2.4 ± 0.5
Serum total cholesterol (mg/dL)^*b^	409 ± 120	320 ± 95	366 ± 124	290 ± 89
Hemoglobin A1c (%)^b^	5.3 ± 0.7	5.5 ± 0.9	5.4 ± 0.9	5.2 ± 0.5
Use of RAS blockers, *N* (%)^*^	21 (13.5)	68 (45.9)	16 (42.1)	15 (45.5)
Use of statins, *N* (%)^*^	41 (27.1)	71 (48.0)	21 (55.3)	5 (15.2)
Use of antidiabetic drugs, *N* (%)^*^	7 (4.5)	4 (2.7)	2 (5.3)	3 (9.1)
Immunosuppressive therapy, *N* (%)^*^	153 (98.7)	127 (85.8)	35 (92.1)	24 (72.7)
Time from kidney biopsy to immunosuppressive therapy (day)	3 (-4, 7)	10 (4, 24)	6 (0, 14)	10 (4, 14)
< 0 day, N (%)	45 (29.0)	14 (9.5)	9 (23.7)	4 (12.1)
Immunosuppressive drugs within 24 months of immunosuppressive therapy, *N* (%)^b^				
Prednisolone	150 (98.7)	118 (95.2)	35 (100.0)	22 (100.0)
Intravenous methylprednisolone	48 (31.6)	26 (21.0)	11 (31.4)	11 (50.0)
Cyclosporine	54 (35.5)	70 (56.5)	22 (62.9)	6 (27.3)
Tacrolimus	2 (1.3)	5 (4.0)	0 (0.0)	0 (0.0)
Cyclophosphamide	1 (0.7)	9 (4.8)	0 (0.0)	3 (13.6)
Mizoribine	8 (5.3)	23 (18.5)	0 (0.0)	1 (4.5)
Mycophenolate mofetil	1 (0.7)	2 (1.6)	0 (0.0)	0 (0.0)
Rituximab	4 (2.6)	1 (0.8)	1 (2.9)	0 (0.0)

Mean ± standard deviation; median (25%, 75)

*Cr* creatinine, *eGFR* estimated glomerular filtration rate, *FSGS* focal segmental glomerulosclerosis, *MCD* minimal change disease, *MN* membranous nephropathy, *RAS* renin-angiotensin system

^*^P < 0.05 for chi-square test, Fisher's exact test, ANOVA, or Kruskal–Wallis test, as appropriate

^a^Baseline visit was set on the date of kidney biopsy or the date of initiating immunosuppressive therapy, whichever came first

^b^Number of missing value: body mass index, *N* = 5 in MCD; systolic and diastolic blood pressure, *N* = 4 in MCD; Urinary protein, *N* = 1 and 1 in MCD and FSGS; eGFR, *N* = 16, 1, and 1 in MCD, MN, and others because of < 18 year of age; serum total cholesterol, *N* = 10, 7, and 3 in MCD, MN, and others; hemoglobin A1c, *N* = 45, 31, 8, and 14 in MCD, MN, FSGS, and others; initial drugs within 1 month of immunosuppressive therapy, *N* = 1, 3, and 2 in MCD, MN, and others

^c^Urinary protein/creatinine ratio (g/gCr) was used in 36 (23.4%), 21 (14.3%), 8 (21.1%), and 6 (18.2%) patients with MCD, MN, FSGS, and others, respectively, who had missing value of urinary protein (g/day)

The majority of patients received immunosuppressive therapy within a median (interquartile range) of 3 (− 4, 7), 10 (4, 24), 6 (0, 14), and 10 (4, 14) days of kidney biopsy in 153 (98.7%), 127 (85.8%), 35 (92.1%), and 24 (72.7%) patients with MCD, MN, FSGS, and others, respectively (Table 1). Almost all patients received prednisolone within 24 months of immunosuppressive therapy (98.7%, 95.2%, 100.0%, and 100.0% in MCD, MN, FSGS, and others). One-third of the patients with MCD additionally received intravenous methylprednisolone (31.6%) and cyclosporine (35.5%) within 24 months of immunosuppressive therapy. In patients with MN and FSGS, cyclosporine (56.5% and 62.9%, respectively) was much more common than intravenous methylprednisolone (21.0% and 31.4%, respectively). In contrast, a half of patients with other glomerulonephritides received intravenous methylprednisolone (50.0%), followed by cyclosporine (27.3%). Other immunosuppressive drugs were rarely used, except mizoribine in patients with MN (18.5%).

**Fig. 2:**
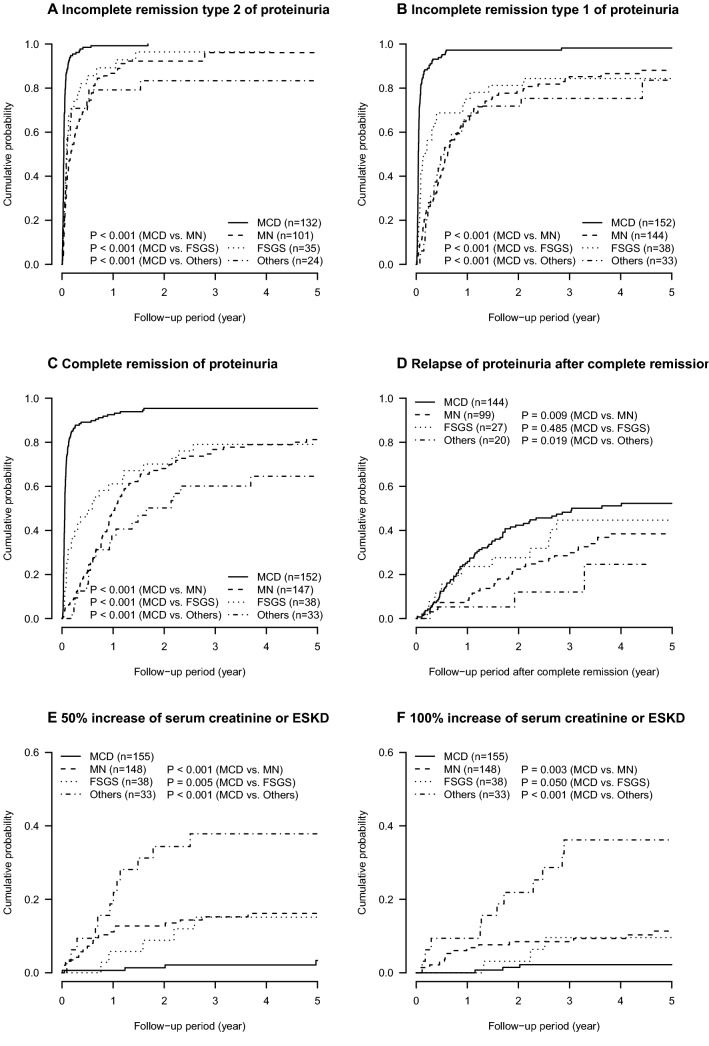

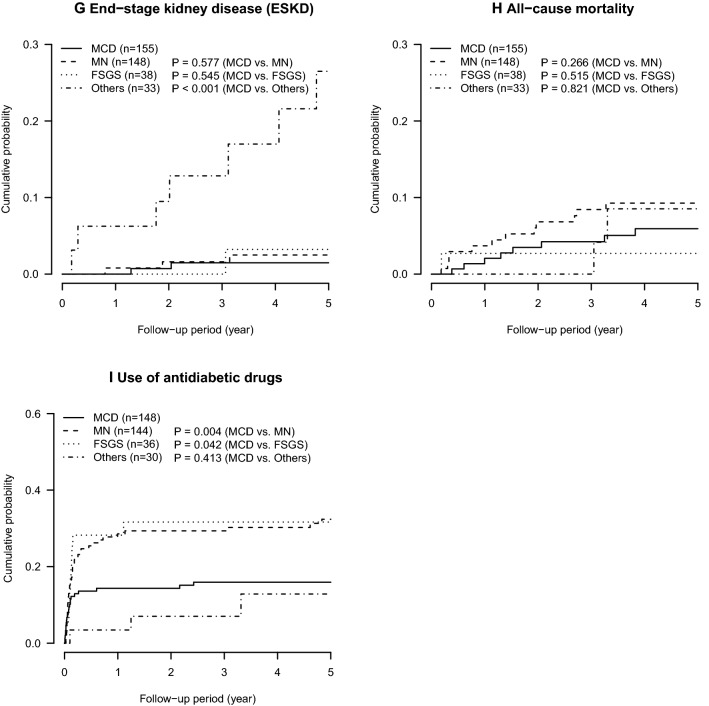
Cumulative probabilities of major clinical outcomes: incomplete remission type 1 (**a**) and 2 (**b**), complete remission (**c**), relapse of proteinuria after complete remission (**d**), 50% and 100% increase in serum creatinine and/or end-stage kidney disease (ESKD) (**e**, **f**), ESKD (**g**), all-cause mortality (**h**), and use of diabetic drugs (**i**)

The cumulative probabilities of remission and relapse of proteinuria during the median follow-up period of 5.0 years (interquartile range 3.2–5.0) are described in Fig. 2a–d. Within one year of the baseline visit, urinary protein decreased below the nephrotic range of proteinuria indicating incomplete remission type 2, in approximately 80% or more of the nephrotic patients at the baseline visit (cumulative probability of incomplete remission type 2 of proteinuria: 0.99 [95% confidence interval 0.95, 1.00], 0.87 [0.78, 0.92], 0.89 [0.69, 0.94], and 0.79 [0.55, 0.90] in patients with MCD, MN, FSGS, and others, respectively) (Table 2). Complete remission of proteinuria (24-h urinary protein of < 0.3 g/day or urinary protein-to-creatinine ratio of < 0.3 g/gCr) was observed in approximately half of the patients with MN and FSGS within one year of the baseline visit. Their cumulative probabilities of complete remission increased by 75% within 3 years of the baseline visit (0.77 [0.68, 0.83] and 0.79 [0.60, 0.89] in patients with MN and FSGS, respectively), whereas only 60% in patients with other glomerulonephritides (0.60 [0.39, 0.74]) (Table 2). Patients with MCD, who had the highest cumulative probability of complete remission, also had the highest cumulative probability of relapse of proteinuria (Table 3). Approximately half of the patients with MCD experienced relapse of proteinuria within 3 years of the baseline visit (0.48 [0.39, 0.56]). Relapse of proteinuria was also common in patients with FSGS (0.44 [0.20, 0.60]). Most patients with MCD and FSGS developed their first relapse during immunosuppressive therapy (79.1% and 90.9%, respectively).

**Table 2:** Incidence of remission of proteinuria in primary nephrotic syndrome

	MCD	MN	FSGS	Others
Incomplete remission type 2 of proteinuria (urinary protein < 3.5 g/day or g/gCr)				
Baseline urinary protein ≥ 3.5 g/day (or g/Cr), *N*	132	101	35	24
Incidence of remission, *N* (%)	132 (100.0)	90 (89.1)	32 (91.6)	20 (83.3)
Time to remission (day)	13 (8, 20)	50 (21, 146)	30 (17, 54)	28 (17, 59)
Cumulative probability of remission (95% CI)				
1 month	0.86 (0.79, 0.91)	0.31 (0.21, 0.40)	0.46 (0.26, 0.60)	0.46 (0.22, 0.63)
2 months	0.94 (0.88, 0.97)	0.48 (0.37, 0.57)	0.69 (0.49, 0.81)	0.67 (0.41, 0.81)
1 year	0.99 (0.95, 1.00)	0.87 (0.78, 0.92)	0.89 (0.69, 0.96)	0.79 (0.55, 0.90)
3 years	NA	0.96 (0.82, 0.99)	0.96 (0.76, 0.99)	0.93 (0.59, 0.93)
Incomplete remission type 1 of proteinuria (urinary protein < 1.0 g/day or g/gCr)				
Baseline urinary protein ≥ 1.0 g/day (or g/Cr), *N*	152	144	38	33
Incidence of remission, *N* (%)	148 (97.4)	108 (75.0)	30 (78.9)	25 (75.8)
Time to remission (day)	15 (11, 25)	178 (56, 316)	33 (23, 111)	148 (75, 252)
Cumulative probability of remission (95% CI)				
1 month	0.77 (0.69, 0.83)	0.09 (0.04, 0.14)	0.32 (0.15, 0.45)	0.06 (0.00, 0.14)
2 months	0.88 (0.82, 0.92)	0.20 (0.13, 0.27)	0.50 (0.31, 0.64)	0.09 (0.00, 0.19)
1 year	0.97 (0.93, 0.99)	0.67 (0.58, 0.75)	0.75 (0.55, 0.86)	0.66 (0.44, 0.79)
3 years	0.98 (0.94, 0.99)	0.85 (0.77, 0.91)	0.84 (0.64, 0.93)	0.75 (0.54, 0.87)
Complete remission of proteinuria (urinary protein < 0.3 g/day or g/gCr)				
Baseline urinary protein ≥ 0.3 g/day (or g/Cr), *N*	152	147	38	33
Incidence of remission, *N* (%)	144 (94.7)	100 (68.0)	28 (73.7)	20 (60.6)
Time to remission (day)	19 (13, 31)	292 (152, 443)	82 (31, 283)	290 (185, 558)
Cumulative probability of remission (95% CI)				
1 month	0.70 (0.62, 0.77)	0.05 (0.01, 0.08)	0.18 (0.05, 0.30)	0.00 (0.00, 0.00)
2 months	0.83 (0.76, 0.88)	0.07 (0.03, 0.11)	0.32 (0.15, 0.45)	0.00 (0.00, 0.00)
1 year	0.93 (0.87, 0.96)	0.48 (0.39, 0.56)	0.61 (0.41, 0.74)	0.37 (0.18, 0.52)
3 years	0.95 (0.90, 0.98)	0.77 (0.68, 0.83)	0.79 (0.60, 0.89)	0.60 (0.39, 0.74)

Median (25%, 75%)

*CI* confidence interval, *Cr* creatinine, *FSGS* focal segmental glomerulosclerosis, *MCD* minimal change disease, *MN* membranous nephropathy, *NA* not assessed

**Table 3:** Incidence of relapse of proteinuria after complete remission in primary nephrotic syndrome

	MCD	MN	FSGS	Others
Follow-up after complete remission > 0 day, *N*	144	99	27	20
Incidence of relapse, *N* (%)	67 (46.5)	33 (33.3)	11 (40.7)	3 (15.0)
Time from complete remission to 1st relapse (year)	0.96 (0.50, 1.69)	1.59 (1.03, 2.59)	0.88 (0.43, 2.40)	1.92 (1.10, 2.60)
Use of immunosuppressive drugs at 1st relapse, *N* (%)				
Prednisolone	47 (70.1)	15 (45.5)	9 (81.8)	2 (66.7)
Cyclosporine	9 (13.4)	8 (24.2)	6 (54.5)	1 (33.3)
Tacrolimus	0 (0.0)	0 (0.0)	0 (0.0)	0 (0.0)
Cyclophosphamide	1 (1.5)	1 (3.0)	0 (0.0)	0 (0.0)
Mizoribine	2 (3.0)	1 (3.0)	0 (0.0)	0 (0.0)
Mycophenolate mofetil	0 (0.0)	0 (0.0)	0 (0.0)	0 (0.0)
Any drugs	53 (79.1)	17 (51.5)	10 (90.9)	2 (66.7)
Cumulative probability of relapse (95% CI)				
1 year	0.26 (0.18, 0.33)	0.08 (0.03, 0.14)	0.24 (0.05, 0.38)	0.05 (0.00, 0.15)
2 years	0.42 (0.33, 0.50)	0.22 (0.13, 0.30)	0.28 (0.08, 0.43)	0.12 (0.00, 0.26)
3 years	0.48 (0.39, 0.56)	0.30 (0.20, 0.39)	0.44 (0.20, 0.60)	0.12 (0.00, 0.26)

Median (25%, 75%)

*CI* confidence interval, *Cr* creatinine, *FSGS* focal segmental glomerulosclerosis, *MCD* minimal change disease, *MN* membranous nephropathy

**Fig. 3:**
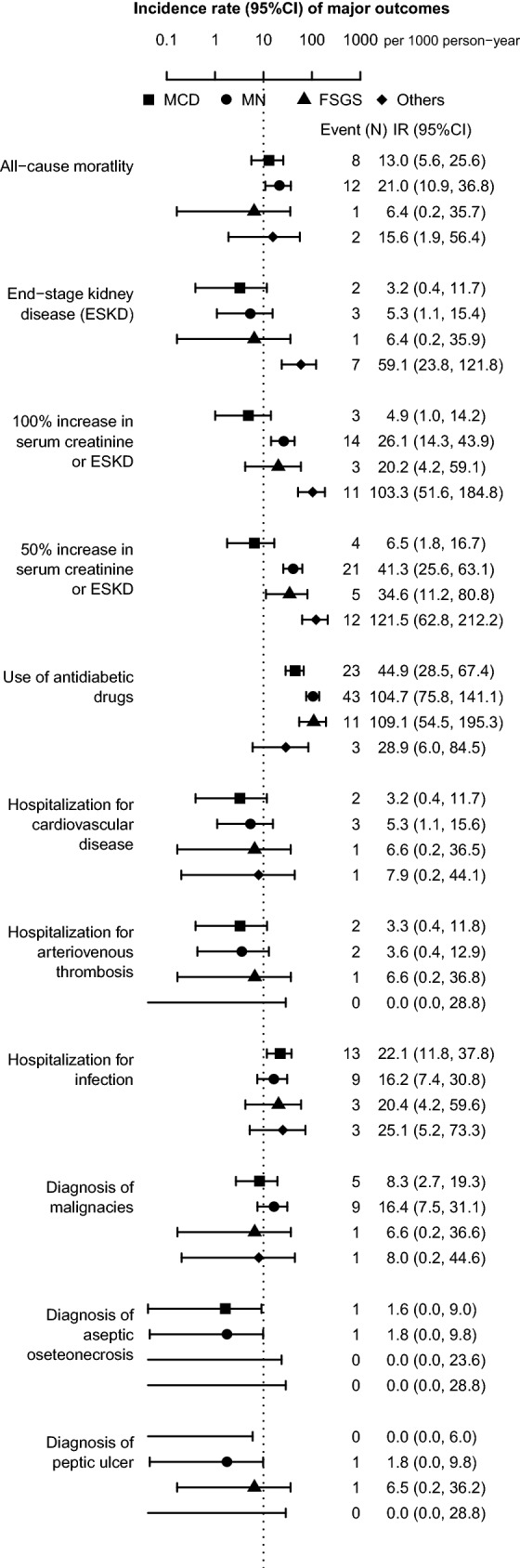
Incidence rates of major outcomes in primary nephrotic syndrome; causes of mortality included infection (*N* = 6, 5, and 1 in MCD, MN, and FSGS, respectively), malignancy (*N* = 1, 5, and 1 in MCD, MN, and other glomerulonephritides, respectively), cardiovascular disease (*N* = 1 and 1 in MCD and MN, respectively) and others (*N* = 1, 1, and 1 in MCD, MN, and other glomerulonephritides, respectively)

Patients with other glomerulonephritides had a higher risk of decrease in GFR, followed by that in patients with MN, FSGS, and MCD (Figs. 2e–g, 3). The incidence rate per 1000 person-years of 50% increase in serum creatinine or ESKD was 6.5 (95% confidence interval, 1.8, 16.7), 41.3 (25.6, 63.1), 34.6 (11.2, 80.8), and 121.5 (62.8, 212.2) in patients with MCD, MN, FSGS, and others, respectively (Fig. 3). Because of a short follow-up period of 5 years, the incidence of ESKD was low in patients with MCD, MN, and FSGS (per 1000 person-years; 3.2 [0.4, 11.7], 5.3 [1.1, 15.4], 6.4 [0.2, 35.9], 59.1 [23.8, 121.8] in MCD, MN, FSGS, and others, respectively).

Compared with ESKD, all-cause mortality was more common in patients with MCD and MN (per 1000 person-years; 13.0 [5.6, 25.6] and 21.0 [10.9, 36.8] in MCD and MN, respectively) (Figs. 2g–h, 3). The leading cause of death was infection (*N* = 11 [47.8%]), followed by malignancy (*N* = 7 [30.4%]), CVD (*N* = 2 [8.7%]) and others (*N* = 3 [13.0%]) (Fig. 3). In patients with MCD and MN, infection was the leading cause of death (*N* = 6 [66.7%] and 5 [41.7%] in MCD and MN, respectively). Although the incidence of hospitalization for infection was comparable among the four groups of glomerulonephritides (per 1000 person-years; 22.1 [11.8, 37.8], 16.2 [7.4, 30.8], 20.4 [4.2, 59.6], 25.1 [5.2, 73.3] in MCD, MN, FSGS, and others, respectively) (Fig. 3), patients with MCD were associated with hospitalization for infection at a marginally significant level than those with MN, after controlling for age and sex (vs. MN; MCD, adjusted hazard ratio 2.41 [95% confidence interval 0.98, 5.94], *P* = 0.06; FSGS, 1.58 [0.43, 5.88], *P* = 0.5; others, 1.78 (0.48, 6.58), *P* = 0.4) (Table 4). After an additional adjustment for serum creatinine, MCD was significantly associated with hospitalization for infection (Model 2: MCD, 2.44 [1.00, 5.95], *P* = 0.05; FSGS, 1.48 [0.40, 5.50], *P* = 0.6; other glomerulonephritides, 1.26 [0.30, 5.29], *P* = 0.6). A further adjustment for urinary protein confirmed their associations (Model 3).

**Table 4:** Incidence of hospitalization for infection in primary nephrotic syndrome

	MCD	MN	FSGS	Others
Incidence of infection, *N* (%)	13 (8.4)	9 (6.1)	3 (7.9)	3 (9.1)
Follow-up period (year)	4.9 (2.8, 5.0)	5.0 (2.7, 5.0)	5.0 (3.9, 5.0)	4.1 (2.6, 5.0)
Incidence rate of infection, per 1000 person-years	22.1 (11.8, 37.8)	16.2 (7.4, 30.8)	20.4 (4.2, 59.6)	25.1 (5.2, 73.3)
Hazard ratio (95% CI)^a^				
Unadjusted model	1.33 (0.57, 3.11)	1.00 (reference)	1.27 (0.35, 4.71)	1.48 (0.40, 5.46)
Multivariable-adjusted model 1	2.41 (0.98, 5.94)^†^	1.00 (reference)	1.58 (0.43, 5.88)	1.78 (0.48, 6.58)
Multivariable-adjusted model 2	2.44 (1.00, 5.95)^‡^	1.00 (reference)	1.48 (0.40, 5.50)	1.26 (0.30, 5.29)
Multivariable-adjusted model 3	2.56 (1.04, 6.34)^‡^	1.00 (reference)	1.55 (0.41, 5.83)	1.22 (0.29, 5.19)

*CI* confidence interval, *FSGS* focal segmental glomerulosclerosis, *MCD* minimal change disease, *MN* membranous nephropathy

^†^*P* = 0.06

^‡^*P* < 0.05

^a^Model 1 adjusted for baseline age and stratified by sex; Model 2 adjusted for baseline age and serum creatinine and stratified by sex; Model 3 adjusted for baseline age, serum creatinine, and urinary protein and stratified by sex

The use of antidiabetic drugs was common especially among patients with MN and FSGS (Fig. 2I). Their cumulative incidence of use of antidiabetic drugs was approximately 30% one year after the baseline visit (1-year cumulative probability in MCD, MN, FSGS, and other glomerulonephritides: 0.14 [0.08, 0.20], 0.28 [0.20, 0.35], 0.28 [0.12, 0.42], and 0.03 [0.00, 0.10], respectively). During the 5-year follow-up period, other clinical outcomes were relatively rare, including hospitalization for malignancy, CVD, thrombosis, aseptic osteonecrosis, and peptic ulcer (Fig. 3).

## Discussion

The present 5-year cohort study, which included 374 patients with primary nephrotic syndrome in 55 hospitals in Japan, clarified the incidence rate of major clinical outcomes and disclosed that the incidence of all-cause mortality was higher than that of ESKD in patients with two major glomerulonephritides, MCD and MN. Hospitalization for infection, the leading cause of all-cause mortality, was significantly more common in patients with MCD than those with MN, suggesting that patients with MCD were vulnerable to infection. Several advantages of the present study include the nature of the cohort study design, the inclusion of two major glomerulonephritides, MCD and MN, the measurements of a wide variety of major clinical outcomes, and the collection of recent real-world clinical data in the most recent decade between 2009 and 2015.

Few studies have compared the incidence rates of ESKD and all-cause mortality among patients with primary nephrotic syndrome. A Korean single-center retrospective cohort study, including 187, 232, and 251 patients with MCD, MN, and FSGS, showed that all-cause mortality was more common than ESKD in MCD and MN during the median observational period of 7.5 years, whereas ESKD was more common than all-cause mortality in FSGS [21]. A similar finding was also reported in a Taiwanese single-center retrospective cohort study with a median observational period of 5.9 years, including 109, 209, and 132 patients with MCD, MN, and FSGS, respectively [22]. However, these studies did not clarify the causes of mortality. After confirming that all-cause mortality was more common than ESKD in patients with MCD and MN, the present multicenter cohort study identified infection as the leading cause of mortality (Fig. 3) and disclosed that patients with MCD were more vulnerable to infection compared to patients with MN (Table 4). One of the plausible reasons for the higher incidence rate of infection in patients with MCD might be due to the higher incidence of relapses of proteinuria with add-on use of immunosuppressive drugs. Compared to patients with MN, patients with MCD had a higher risk of relapse of proteinuria during immunosuppressive therapy (Table 3), probably leading to the higher doses of immunosuppressive drugs. Unfortunately, the dose of each immunosuppressive drug during the immunosuppressive therapy was not available in the present study. Further research with details of immunosuppressive drugs are essential to assess an association between immunosuppressive therapy and infection.

Comparable with the results of the present cohort study, previous retrospective cohort studies on MCD reported that infection was one of the most common adverse events [23–27] and one of the leading causes of mortality [28]. To suppress the incidence of infection, a lower dose of and/or shorter term immunosuppressive therapy is desirable. Among pediatric patients with corticosteroid-sensitive nephrotic syndrome, two randomized trials in Japan [29] and India [30] recently demonstrated that the incidence of relapse of proteinuria was comparable between conventional 6-month corticosteroid therapy and 3-month corticosteroid therapy. In adult patients with MCD, only low-quality evidences in this regard are available. Several guidelines suggested longer corticosteroid therapy; the Kidney Disease Improving Global Outcomes (KDIGO) clinical guideline for glomerulonephritis suggested a daily dose of 1.0 mg/kg of prednisolone or an alternate-day single dose of 2 mg/kg tapered slowly over a total period of up to 6 months [12] and the Japanese evidence-based clinical practice guideline for nephrotic syndrome suggest 0.6–0.8 mg/kg of prednisolone tapered within 2 years [13]. Compared with these conventional long corticosteroid therapies, an intriguing Japanese observational study of adult patients with MCD suggested clinical advantages of a 2-month corticosteroid therapy, the lower incidence of adverse events, including diabetes and infection [31]. Because nephrologists might possibly maintain adult patients with MCD on corticosteroids for very long [32], an optimal immunosuppressive therapy should be explored in well-designed clinical studies to prevent critical events associated with immunosuppressive therapy, including infection.

The present study has several limitations. First, the incidence of all-cause mortality (*N* = 22) and ESKD (*N* = 12) was small; thus, the findings of the present study might not be reproducible. The JNSCS is planning to extend the 5-year follow-up period to 10 years, providing more precise details regarding the clinical impacts of major outcomes in patients with primary nephrotic syndrome. Second, the small number of patents with FSGS and other glomerulonephritides hindered statistically meaningful analyses in JNSCS. The results of the present study suggested the cumulative probabilities of complete remission of proteinuria and an increase of serum creatinine of FSGS were comparable to those of MN (Fig. 2c, e, f), although the higher rate of relapse of proteinuria of FSGS, which was comparable to that of MCD (Fig. 2d). Because the higher probability of relapse might contribute to the higher risk of infection in patients with MCD, those with FSGS might be similarly vulnerable to infection. To clarify their clinical courses, a larger cohort study is needed. Third, the incidences of some outcomes were dependent on the practice patterns of each hospital. For example, the thresholds of plasma glucose concentration and/or hemoglobin A1c to start antidiabetic drugs might be different among the hospitals.

In conclusion, the JNSCS revealed that patients with MCD and MN had a higher risk of all-cause mortality than that of ESKD. Patients with MCD were more vulnerable to infection, the leading cause of mortality, compared to patients with MN. These results provide pivotal information that identifies the treatment goals of primary nephrotic syndrome with the recent immunosuppressive therapy. Nephrologists might possibly pay more attention to infection in patients with primary nephrotic syndrome.

## Electronic supplementary material

Below is the link to the electronic supplementary material.

Supplementary file1 (PDF 88 kb)
