# Supplementary material for: Incidence of remission and relapse of proteinuria, end-stage kidney disease, mortality, and major outcomes in primary nephrotic syndrome: the Japan Nephrotic Syndrome Cohort Study (JNSCS)
Source: Clin Exp Nephrol. 2020 Mar 7;24(6):526–40. doi: 10.1007/s10157-020-01864-1 (PMC7248042; doi:10.1007/s10157-020-01864-1)

**Supplemental Material**

**Supplementary figure 1.** Flow diagram of 374 patients enrolled in JNSCS for incidences of major clinical outcomes: (A) remission and relapse of proteinuria, 50% and 100% increase in serum creatinine, end-stage kidney disease, and all-cause mortality; (B) use of diabetic drugs, diagnosis of malignancy, aseptic osteonecrosis, and peptic ulcer, and hospitalization for infection, cardiovascular disease, and arteriovenous thrombosis.

Supplementary figure 1A

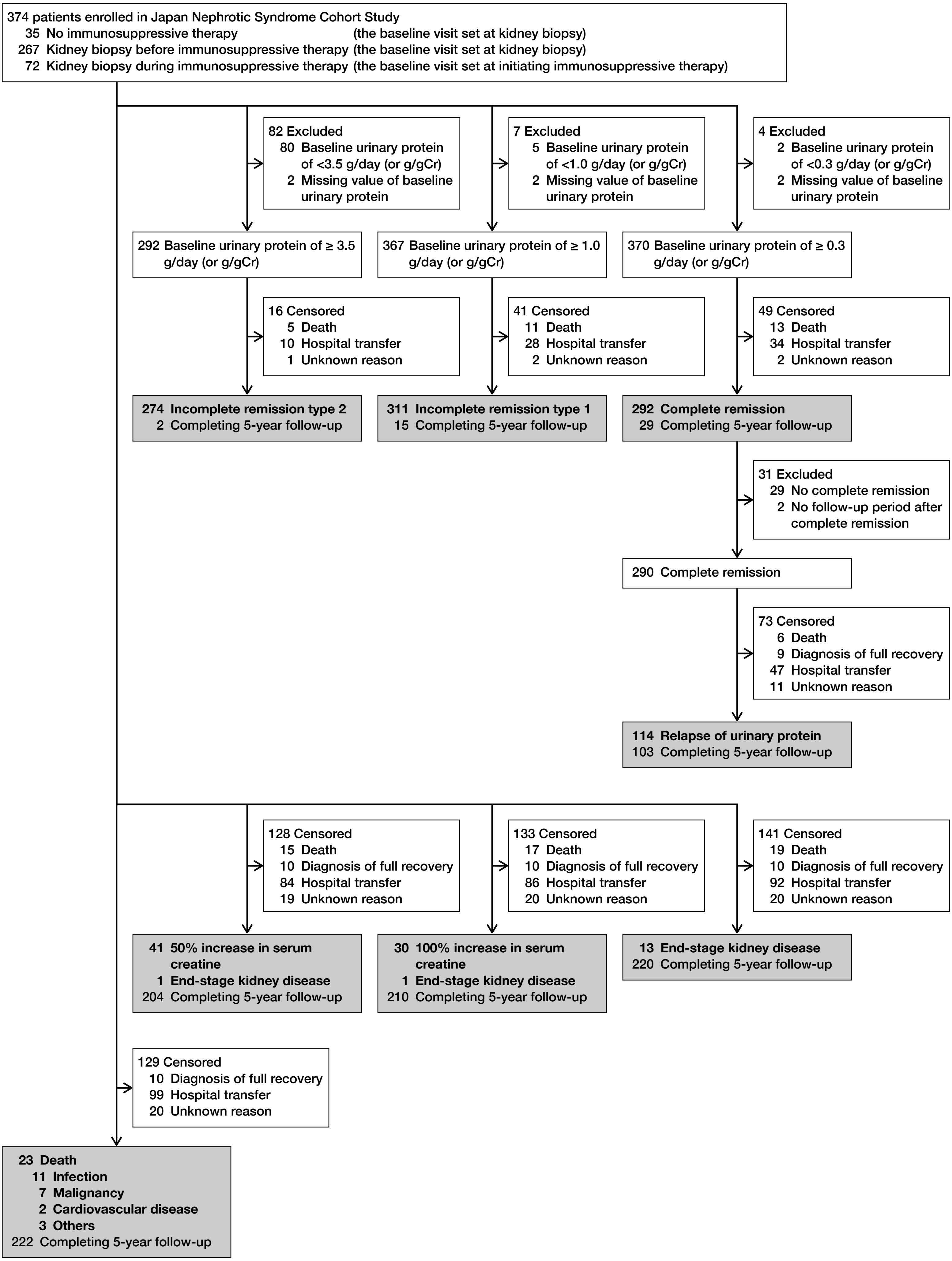

Supplementary figure 1B

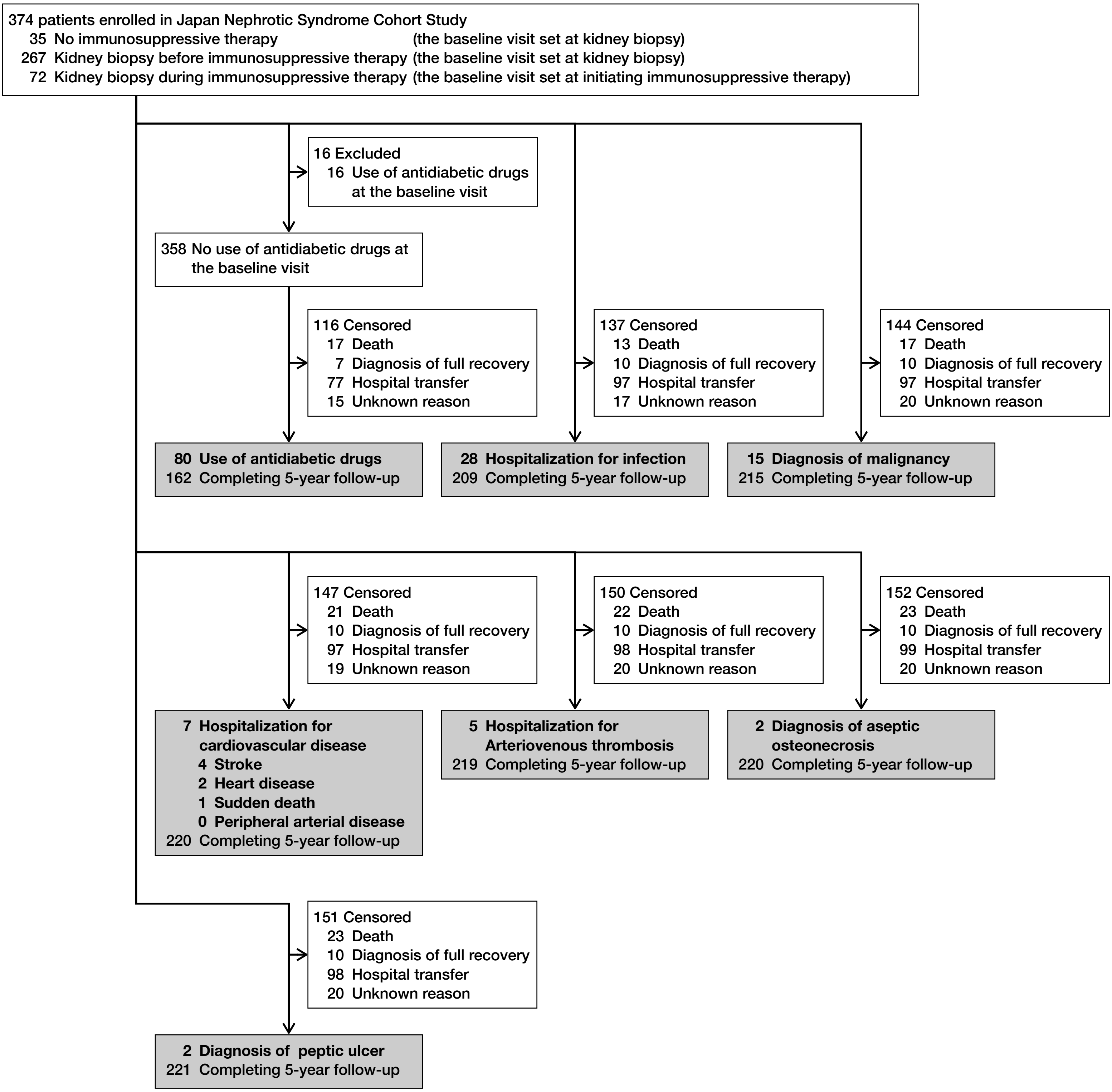

Supplement: Supplementary file 1 — Supplementary file1 (PDF 88 kb) [file 10157_2020_1864_MOESM1_ESM.pdf]
